# Renal Hemodynamics During Sympathetic Activation Following Aerobic and Anaerobic Exercise

**DOI:** 10.3389/fphys.2018.01928

**Published:** 2019-01-10

**Authors:** Zachary J. Schlader, Christopher L. Chapman, Julia M. Benati, Elizabeth A. Gideon, Nicole T. Vargas, Penelope C. Lema, Blair D. Johnson

**Affiliations:** ^1^Center for Research and Education in Special Environments, Department of Exercise and Nutrition Sciences, University at Buffalo, Buffalo, NY, United States; ^2^Department of Emergency Medicine, Jacobs School of Medicine and Biomedical Sciences, University at Buffalo, Buffalo, NY, United States

**Keywords:** renal vascular resistance, cold pressor test, face cooling, Doppler ultrasound, exercise recovery

## Abstract

We tested the hypotheses that prior aerobic (Study 1) or anaerobic (Study 2) exercise attenuates the increase in renal vascular resistance (RVR) during sympathetic stimulation. Ten healthy young adults (5 females) participated in both Study 1 (aerobic exercise) and Study 2 (anaerobic exercise). In Study 1, subjects completed three minutes of face cooling pre- and post- 30 min of moderate intensity aerobic exercise (68 ± 1% estimate maximal heart rate). In Study 2, subjects completed two minutes of the cold pressor test pre- and post- the completion of a 30 s maximal effort cycling test (Wingate Anaerobic Test). Both face cooling and the cold pressor test stimulate the sympathetic nervous system and elevate RVR. The primary dependent variable in both Studies was renal blood velocity, which was measured at baseline and every minute during sympathetic stimulation. Renal blood velocity was measured via the coronal approach at the distal segment of the right renal artery with pulsed wave Doppler ultrasound. RVR was calculated from the quotient of mean arterial pressure and renal blood velocity. In Study 1, renal blood velocity and RVR did not differ between pre- and post- aerobic exercise (*P* ≥ 0.24). Face cooling decreased renal blood velocity (*P* < 0.01) and the magnitude of this decrease did not differ between pre- and post- aerobic exercise (*P* = 0.52). RVR increased with face cooling (*P* < 0.01) and the extent of these increases did not differ between pre- and post- aerobic exercise (*P* = 0.74). In Study 2, renal blood velocity was 2 ± 2 cm/s lower post- anaerobic exercise (*P* = 0.02), but RVR did not differ (*P* = 0.08). The cold pressor test decreased renal blood velocity (*P* < 0.01) and the magnitude of this decrease did not differ between pre- and post- anaerobic exercise (*P* = 0.26). RVR increased with the cold pressor test (*P* < 0.01) and the extent of these increases did not differ between pre- and post- anaerobic exercise (*P* = 0.12). These data indicate that 30 min of moderate intensity aerobic exercise or 30 s of maximal effort anaerobic exercise does not affect the capacity to increase RVR during sympathetic stimulation following exercise.

## Introduction

Blood pressure is often reduced for up to 60 min following an acute bout of dynamic exercise (Halliwill et al., 2013, 2014). In healthy untrained adults, this post-exercise hypotension is caused by reductions in total peripheral resistance (TPR) that are not fully offset by increases in cardiac output (Meade et al., 2018). Renal, splanchnic and cutaneous vascular resistances return to pre- exercise levels within ∼20 min following exercise (Pricher et al., 2004; Wilkins et al., 2004). Thus, the lower TPR is due to persistent vasodilation in the muscle vasculature following exercise (Halliwill et al., 2013, 2014).

Orthostatic tolerance, defined as the ability to maintain blood pressure during orthostasis (Schlader et al., 2016b), is often impaired following dynamic exercise (Halliwill et al., 2014). This is caused by a relative inability to maintain stroke volume during orthostasis, which is likely due to inadequate increases in resistance in the muscle vasculature, thereby promoting pooling of blood in this vascular bed (Halliwill et al., 2013, 2014). That said, orthostasis also provokes sympathetically mediated increases in resistance in the splanchnic (Rowell et al., 1972; Jarvis et al., 2012) and renal (Bakris et al., 1986; Hirsch et al., 1989; Minson et al., 1999) vasculatures. These vasculatures each receive 20–30% of cardiac output and contribute significantly to blood pressure regulation during orthostasis (Rowell et al., 1972; Hirsch et al., 1989). For instance, pharmacological redistribution of blood flow away from the splanchnic vasculature by ∼140 mL/min (or ∼3% of cardiac output) improves orthostatic tolerance (Jarvis et al., 2012). Moreover, orthostatic stress reduces renal blood flow by a similar magnitude (∼170 mL/min) (Hirsch et al., 1989). Thus, despite resistance in these visceral vascular beds returning to pre-exercise levels shortly after exercise (Pricher et al., 2004), it may be that prior exercise attenuates the ability to increase vascular resistance during sympathetic activation. If this were the case, the renal and/or splanchnic vasculatures could contribute to post-exercise orthostatic intolerance. However, the effect of prior exercise on the hemodynamic response to sympathetic stimulation in one of these visceral vasculatures is unknown.

With this background, the purpose of this study was to test the hypothesis that prior aerobic exercise attenuates the increase in renal vascular resistance (RVR) during sympathetic stimulation. The extent of post-exercise orthostatic intolerance is influenced by the intensity of the exercise (Mündel et al., 2015). Moreover, there is indirect evidence that high intensity (mostly anaerobic) exercise elicits a greater incidence of pre-syncope (i.e., the onset of syncopal signs and symptoms) during orthostasis compared to moderate intensity (primarily aerobic) exercise (Halliwill et al., 2014). Furthermore, reductions in renal blood flow can be maintained for up to 60 min following high intensity exercise (Suzuki et al., 1996), which is suggestive of sustained alterations in renal vascular control. Thus, we also conducted a separate study that tested the hypothesis that prior anaerobic exercise attenuates the increase in RVR during sympathetic stimulation.

## Materials and Methods

### Ethics Statement

This study was approved by the Institutional Review Board at the University at Buffalo and conformed to the standards set by the Declaration of Helsinki, except for registration in a database. Before completing any study related activities, each subject was fully informed of the experimental procedures and possible risks before giving informed, written consent.

### Subjects

Ten healthy young adults (five females) participated in both Study 1 (aerobic exercise) and Study 2 (anaerobic exercise). Only four subjects completed both Studies. Thus, these Studies were considered independent of one another. The subject characteristics were as follows. Study 1 – age: 23 ± 2 years, height: 169 ± 11 cm, weight: 67.1 ± 14.8 kg; Study 2 – age: 23 ± 3 years, height: 171 ± 11 cm, weight: 69.5 ± 14.5 kg. Subjects were physically active, non-smokers, not taking medications, and reported to be free from any known cardiovascular, metabolic, renal, or neurological diseases. Female subjects were not pregnant, which was confirmed via a urine pregnancy test, and self-reported to be normally menstruating. For both Studies, subjects visited the laboratory on two occasions. Visit one was a screening and familiarization visit, and visit two was the experimental trial. For visit two, subjects arrived at the laboratory having refrained from strenuous exercise, alcohol and caffeine for 12 h, and food for 2 h, and were instructed to be well hydrated. Females were tested throughout their menstrual cycle. This was deemed acceptable because of the repeated measures design and that all experimental testing was conducted on the same day, with shifts in menstrual cycle hormones between the pre- and post- exercise data collection periods likely being minimal.

### Instrumentation and Measurements

Height and weight were measured with a stadiometer and scale (Sartorius Corp. Bohemia, NY, United States). Heart rate was continually measured via a 3-lead ECG (DA100C, Biopac Systems, Inc., Goleta, CA, United States) during experimental testing pre- and post-exercise. During exercise, heart rate was measured using a wireless heart rate monitor (Polar, Kempele, Finland). Beat-to-beat blood pressure was measured via the Penaz method (Finometer Pro, FMS, Amsterdam, Netherlands). These data were confirmed intermittently via auscultation of the brachial artery by electrosphygmomanometry (Tango M2, SunTech Raleigh, NC, United States). In most instances no corrections were necessary, except in one subject in Study 2. In this subject, blood pressure data obtained via auscultation of the brachial artery were used. Stroke volume was estimated from the beat-to-beat blood pressure waveform using Modelflow (Wesseling et al., 1993) (*n* = 9 in Study 2, see above). The partial pressure of end-tidal carbon dioxide (PETCO_2_) was measured via capnography (Nonin Medical, Inc., Plymouth, MN, United States). Due to technical issues, PETCO_2_ was measured in only eight subjects in Study 1. Renal blood velocity was assessed in the distal segment of the right renal artery during the same phase of the respiratory cycle via Doppler ultrasound (GE Vivid 7 Dimension, Chicago, IL, United States) (Momen et al., 2004; Wilson et al., 2007; Drew et al., 2013; Patel et al., 2013). The coronal approach was utilized using a phased-array transducer with a 2.5–3.5 MHz pulsed frequency with subjects in the left lateral recumbent position. The focal zone was set to the artery’s depth, and the transducer was held in the same location for all measurements (Momen et al., 2004; Wilson et al., 2007; Drew et al., 2013; Patel et al., 2013). All measurements and analyses were obtained by the same sonographer (JMB). The transducer location was marked with indelible ink during pre-exercise data collection, which ensured that during post-exercise data collection renal blood velocity measurements were made in the same anatomical position. The insonation angle was always <60° and was the same pre- and post-exercise. Using this approach, the within-subject test–retest coefficient of variation for mean renal blood velocity for the sonographer was 3.9 ± 2.4%. Mean renal blood velocity was indexed by the time-averaged maximum velocity from the envelope of the velocity waveform. At each measurement period, mean renal blood velocity, peak systolic blood velocity and end diastolic blood velocity were measured and averaged over 2–4 cardiac cycles (Momen et al., 2003). Given the depth of the renal artery, it is not possible to accurately measure artery diameter. However, the diameter of the renal artery does not change during pharmacologically induced renal vasoconstriction (Marraccini et al., 1996). Thus, changes in renal blood velocity were interpreted to reflect changes in renal blood flow, as has been done previously (Wilson et al., 2007; Patel et al., 2013; Drew et al., 2017). Renal blood velocity was obtained in all subjects, except one subject in Study 2, where a post-exercise Doppler ultrasound image was not able to be obtained. Therefore, in Study 2 renal blood velocity data are presented as *n* = 9.

### Experimental Protocol

#### Study 1 – Effect of Prior Aerobic Exercise

Following instrumentation, subjects assumed the supine position. Following 10 min of quiet rest, baseline measurements were taken over the next 5 min. At the end of this period, face cooling commenced. Face cooling was achieved by placing a flexible bag of ice water (0°C) directly on the forehead, eyes, and cheeks for 3 min. The volume of the ice water was 2.5 L. Based on previous work from our laboratory (Schlader et al., 2016a, 2018; Johnson et al., 2018), 3 min of face cooling was deemed sufficient to elicit sympathetically mediated increases in vascular resistance and blood pressure, which occurs subsequent to stimulation of trigeminal afferents (Schuitema and Holm, 1988). Moreover, a similar face cooling procedure has been shown to elicit increases in RVR as measured using Doppler ultrasound (Patel et al., 2013). Renal blood velocity data were collected at baseline, each minute of face cooling, and 60 s following face cooling (recovery). These face cooling procedures were completed prior to and following 30 min of aerobic exercise on a treadmill (Quinton Instruments, Seattle, WA, United States) at a speed and grade that elicted a heart rate of 134 ± 3 bpm (68 ± 1% estimate maximal heart rate). All data were collected in a temperature-controlled laboratory (24 ± 2°C, 20 ± 5% relative humidity). The time delay between the end of exercise and the start of the 10 min post- aerobic exercise baseline period was <5 min. This delay was necessary to accommodate re-instrumentation following exercise.

#### Study 2 – Effect of Prior Anaerobic Exercise

Following instrumentation, subjects assumed the supine position. Following 10 min of quiet rest, baseline measurements were taken over the next 5 min. At the end of this period, the cold pressor test commenced. The cold pressor test was achieved by submerging the subject’s right hand in agitated ice water (0°C) up to the wrist for 2 min. This two min cold pressor test elicits sympathetically mediated increases in vascular resistance and blood pressure (Victor et al., 1987; Cui et al., 2010), which occurs subsequent to stimulation of nociceptors (Kregel et al., 1992). Moreover, the cold pressor test has been shown to elicit increases in RVR as measured using Doppler ultrasound (Patel et al., 2013). Renal blood velocity data were collected at baseline, each minute of the cold pressor test, and 60 s following the cold pressor test (recovery). These cold pressor test procedures were completed prior to and following a 30 s Wingate Anaerobic test on a cycle ergometer (Monark 894E, Sweden). This test utilizes mostly anaerobic fuel sources (Beneke et al., 2002). Following a 5 min self-selected warmup, the Wingate Anaerobic Test consisted of a maximal effort against a resistance of 7.5% total body mass following a 3 s unweighted acceleration phase. All data were collected in a temperature-controlled laboratory (23 ± 2°C, 21 ± 13% relative humidity). The time delay between the end of exercise and the start of the 10 min post- anaerobic exercise baseline period was <5 min. This delay was necessary to accommodate re-instrumentation following exercise.

It should be noted that Study 1 and Study 2 were initially designed to test different hypotheses than those of the present studies. As a result, the two Studies used different sympathoexcitatory stimuli and a mostly different cohort of subjects. Despite these differences, however, the stimuli used in Study 1 (face cooling) and Study 2 (cold pressor test) elicit sympathetically mediated increases in RVR (Patel et al., 2013) and the time course of testing both pre- and post-exercise was identical between the two Studies. Thus, the data obtained from these Studies were deemed appropriate to test our hypotheses. That said, given the differences in study design, no direct comparisons were made between the two Studies.

### Data and Statistical Analyses

All non-ultrasound data were sampled continuously at 1000 Hz via a data acquisition system (Biopac MP150, Goleta, CA, United States) in both Studies. These data were binned as a 60 s average at the end of the baseline data collection period, and during and following face cooling (Study 1) or the cold pressor test (Study 2) the non-ultrasound data were binned as a 30 s average every 60 s. Cardiac output was calculated as the product of stroke volume and heart rate, while TPR was calculated as the quotient of mean arterial pressure and cardiac output. RVR was estimated as the quotient of mean arterial pressure and mean renal blood velocity.

To isolate the effect of prior exercise on the responsiveness to face cooling (Study 1) or the cold pressor test (Study 2), data were analyzed as the absolute change from baseline. These data were analyzed using two-way repeated measures ANOVA (time × exercise). When an ANOVA revealed a significant main effect or interaction, *post hoc* Sidak test pairwise comparisons were made. Absolute data at baseline pre- and post- exercise were analyzed using paired *t*-tests. All data were analyzed using Prism software (Version 7, GraphPad Software Inc., La Jolla, CA, United States). *A priori* statistical significance was set at *P* ≤ 0.05 and actual *P*-values are reported where possible. Data are reported as mean ± SD.

## Results

### Study 1 – Effect of Prior Aerobic Exercise

Heart rate was 9 ± 9 bpm higher following exercise (*P* < 0.01), but stroke volume did not differ between pre- and post-exercise (*P* = 0.28, Table 1). As a result, cardiac output was 1.3 ± 1.9 L/min higher following exercise (*P* = 0.03, Table 1). Mean arterial pressure, systolic blood pressure, and diastolic blood pressure were not different between pre- and post-exercise (*P* ≥ 0.08, Table 1). However, TPR was 3.1 ± 4.5 mmHg/L/min lower following exercise (*P* = 0.03, Table 1). PETCO_2_ did not differ between pre- and post-exercise (*P* = 0.26, Table 1). Mean renal blood velocity, peak systolic renal blood velocity, end diastolic renal blood velocity, and RVR did not differ between pre- and post-exercise (*P* ≥ 0.24, Table 1).

**Table 1:** Hemodynamics at baseline pre- and post- aerobic exercise.

	Pre-exercise	Post-exercise	*P*-value
Heart rate (bpm)	60 ± 8	68 ± 7	<0.01
Stroke volume (mL)	83 ± 16	87 ± 23	0.27
Cardiac output (L/min)	4.9 ± 1.0	6.2 ± 2.2	0.03
Mean arterial pressure (mmHg)	85 ± 12	83 ± 9	0.28
Systolic blood pressure (mmHg)	118 ± 17	111 ± 13	0.08
Diastolic blood pressure (mmHg)	65 ± 8	64 ± 8	0.50
TPR (mmHg/L/min)	17.8 ± 3.5	14.7 ± 4.4	0.03
PETCO_2_ (mmHg) (*n* = 8)	41 ± 3	40 ± 1	0.26
Mean renal blood velocity (cm/s)	34 ± 5	34 ± 9	0.31
Peak systolic renal blood velocity (cm/s)	64 ± 10	62 ± 15	0.24
End diastolic renal blood velocity (cm/s)	19 ± 3	20 ± 6	0.26
RVR (mmHg/cm/s)	2.6 ± 0.5	2.6 ± 0.9	0.47

*TPR: total peripheral resistance, PETCO_2_: partial pressure of end tidal carbon dioxide, RVR: renal vascular resistance. Data are presented as Mean ± SD, n = 10 unless noted otherwise.*

**FIGURE 1:**
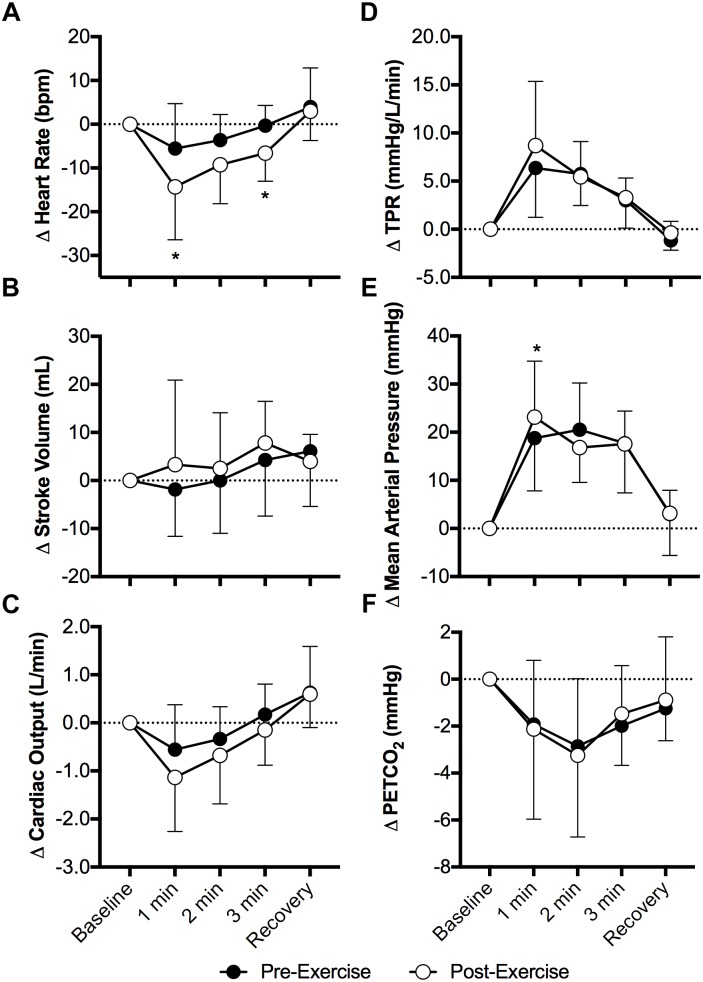
Changes (Δ) in heart rate **(A)**, stroke volume **(B)**, cardiac output **(C)**, total peripheral resistance (TPR, **D**), mean arterial pressure **(E)**, and the partial pressure of end tidal carbon dioxide (PETCO_2_, **F**, *n* = 8) during face cooling pre- and post- aerobic exercise (Study 1). Mean ± SD, *n* = 10 unless noted otherwise. ^∗^different from pre-exercise (*P* ≤ 0.05).

Face cooling decreased heart rate both pre- and post-exercise (*P* < 0.01) and the magnitude of this decrease was greater post-exercise at 1 and 3 min of face cooling (*P* ≤ 0.04, Figure 1A). Stroke volume was not affected by face cooling (*P* = 0.14) and there was no effect of prior exercise (*P* = 0.55, Figure 1B). Cardiac output decreased during face cooling (*P* < 0.01), but the magnitude of this reduction did not differ between pre- and post-exercise (*P* = 0.36, Figure 1C). TPR increased with face cooling (*P* < 0.01) and the magnitude of the increase did not differ between pre- and post-exercise (*P* = 0.47, Figure 1D). Mean arterial pressure increased with face cooling both pre- and post-exercise (*P* < 0.01) and the magnitude of this increase was 4 ± 8 mmHg greater at 1 min post-exercise (*P* = 0.05, Figure 1E). Changes in systolic and diastolic blood pressure mirrored that of mean arterial pressure, with systolic and diastolic blood pressures increasing with face cooling (*P* < 0.01) and the magnitude of these increases being greater at 1 min post-exercise (*P* ≤ 0.05). PETCO_2_ decreased during face cooling (*P* < 0.01) and the extent of these reductions did not differ between pre- and post-exercise (*P* = 0.93, Figure 1F).

**FIGURE 2:**
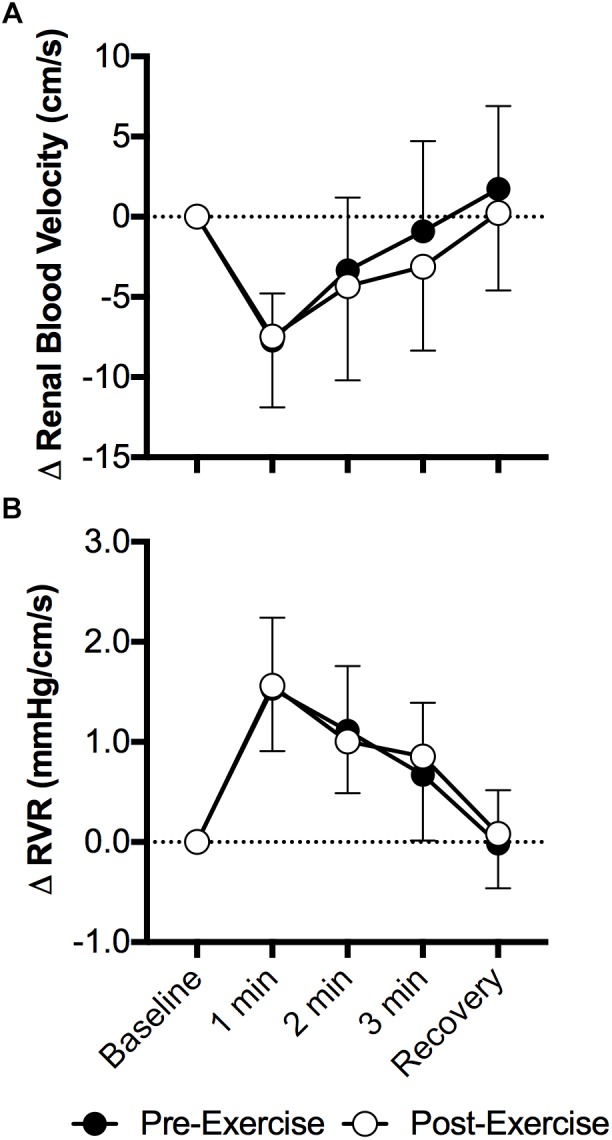
Changes (Δ) in mean renal blood velocity **(A)** and RVR **(B)** during face cooling pre- and post- aerobic exercise (Study 1). Mean ± SD, *n* = 10. Changes in renal blood velocity **(A)** and RVR **(B)** did not differ over time between pre- and post- exercise (interaction: *P* ≥ 0.61).

Face cooling decreased mean renal blood velocity (*P* < 0.01) and the magnitude of this decrease did not differ between pre- and post-exercise (*P* = 0.52, Figure 2A). Changes in peak systolic and end diastolic renal blood velocity mirrored that of mean renal blood velocity, with peak systolic and end diastolic renal blood velocity decreasing with face cooling (*P* < 0.01) and the magnitude of these decreases did not differ between pre- and post-exercise (*P* ≥ 0.27). RVR increased with face cooling (*P* < 0.01) and the extent of these increases did not differ between pre- and post-exercise (*P* = 0.74, Figure 2B).

### Study 2 – Effect of Prior Anaerobic Exercise

Heart rate was 26 ± 12 bpm higher following exercise (*P* < 0.01), but stroke volume did not differ between pre- and post-exercise (*P* = 0.43, Table 2). As a result, cardiac output was 2.3 ± 2.3 L/min higher following exercise (*P* < 0.01, Table 2). Mean arterial pressure, systolic blood pressure, and diastolic blood pressure were not different between pre- and post-exercise (*P* ≥ 0.29, Table 2). However, TPR was 5.2 ± 10.2 mmHg/L/min lower following exercise (*P* = 0.05, Table 2). PETCO_2_ was 6 ± 4 mmHg lower following exercise (*P* < 0.01, Table 2). Mean renal blood velocity was 2 ± 2 cm/s lower post-exercise (*P* = 0.02, Table 2). Peak systolic renal blood velocity was higher post-exercise (by 9 ± 2 cm/s, *P* < 0.01), but end diastolic renal blood velocity was 3 ± 4 cm/s lower post-exercise (*P* < 0.01, Table 2). RVR was not different between pre- and post-exercise (*P* = 0.08, Table 2).

**Table 2:** Hemodynamics at baseline pre- and post- anaerobic exercise.

	Pre-exercise	Post-exercise	*P*-value
Heart rate (bpm)	61 ± 5	87 ± 13	<0.01
Stroke volume (mL) (*n* = 9)	85 ± 22	86 ± 16	0.43
Cardiac output (L/min) (*n* = 9)	5.2 ± 1.6	7.8 ± 2.5	<0.01
Mean arterial pressure (mmHg)	82 ± 13	82 ± 13	0.50
Systolic blood pressure (mmHg)	111 ± 13	114 ± 17	0.29
Diastolic blood pressure (mmHg)	63 ± 15	64 ± 13	0.41
TPR (mmHg/L/min) (*n* = 9)	17.7 ± 6.7	12.1 ± 6.0	0.05
PETCO_2_ (mmHg)	38 ± 4	33 ± 4	<0.01
Mean renal blood velocity (cm/s) (*n* = 9)	34 ± 7	32 ± 7	0.02
Peak systolic renal blood velocity (cm/s) (*n* = 9)	66 ± 13	77 ± 17	<0.01
End diastolic renal blood velocity (cm/s) (*n* = 9)	19 ± 5	15 ± 3	<0.01
RVR (mmHg/cm/s) (*n* = 9)	2.4 ± 0.5	2.6 ± 0.7	0.08

*TPR: total peripheral resistance, PETCO_2_: partial pressure of end tidal carbon dioxide, RVR: renal vascular resistance. Data are presented as Mean ± SD, n = 10 unless noted otherwise.*

**FIGURE 3:**
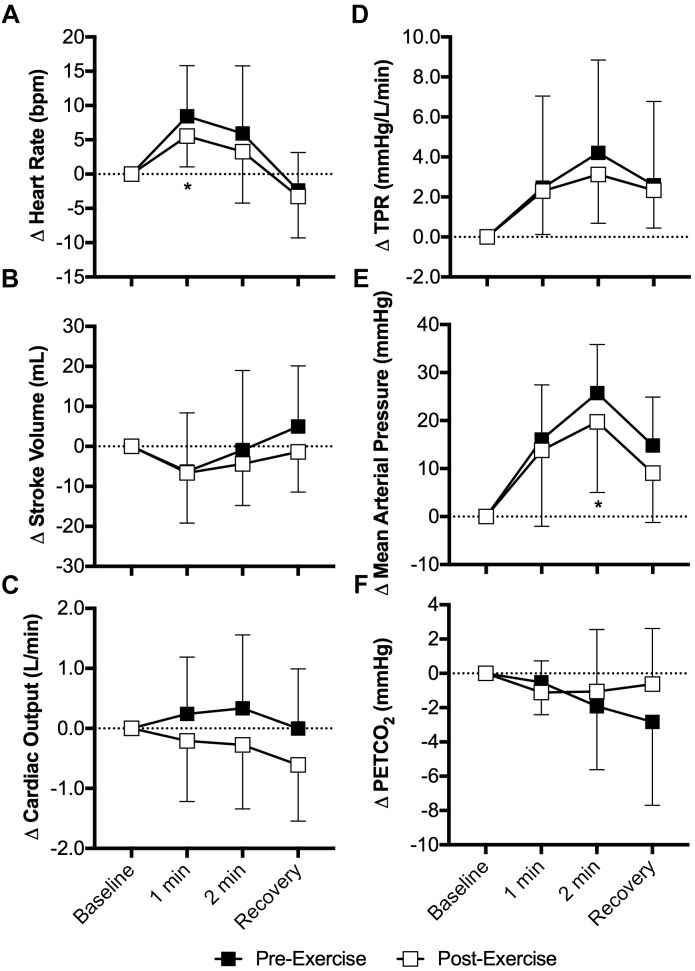
Changes (Δ) in heart rate **(A)**, stroke volume (**B**, *n* = 9), cardiac output (**C**, *n* = 9), TPR (**D**, *n* = 9), mean arterial pressure **(E)**, and the PETCO_2_ **(F)** during the cold pressor test pre- and post- anaerobic exercise (Study 2). Mean ± SD, *n* = 10 unless noted otherwise. ^∗^different from pre-exercise (*P* ≤ 0.05).

Heart rate increased during the cold pressor test both pre- and post-exercise (*P* < 0.01) and the magnitude of this increase was attenuated post-exercise at 1 min of the cold pressor test (*P* = 0.04, Figure 3A). Stroke volume (Figure 3B) and cardiac output (Figure 3C) were not affected by the cold pressor test (*P* ≥ 0.10) and there was no effect of prior exercise (*P* ≥ 0.14). TPR increased during the cold pressor test (*P* < 0.01) and the magnitude of the increase did not differ between pre- and post-exercise (*P* = 0.68, Figure 3D). Mean arterial pressure increased during the cold pressor test both pre- and post-exercise (*P* < 0.01) and the magnitude of this increase was 6 ± 10 mmHg lower at 2 min post-exercise (*P* = 0.05, Figure 3E). Both systolic and diastolic blood pressure increased during the cold pressor test (*P* < 0.01) and the magnitude of these changes did not differ between pre- and post-exercise (*P* ≥ 0.20). PETCO_2_ did not change during the cold pressor test (*P* = 0.20) and there was no effect of prior exercise (*P* = 0.41, Figure 3F).

**FIGURE 4:**
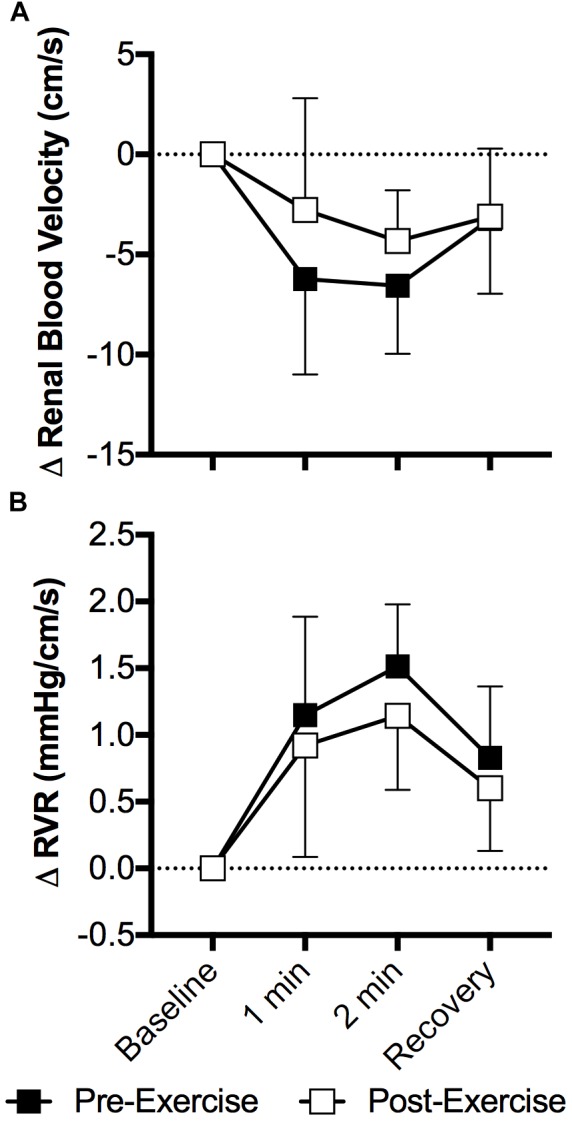
Changes (Δ) in mean renal blood velocity **(A)** and RVR **(B)** during the cold pressor test pre- and post- anaerobic exercise (Study 2). Mean ± SD, *n* = 9. Changes in renal blood velocity **(A)** and RVR **(B)** did not differ over time between pre- and post- exercise (interaction: *P* ≥ 0.39).

The cold pressor test decreased mean renal blood velocity (*P* < 0.01) and the magnitude of this decrease did not differ between pre- and post-exercise (*P* = 0.26, Figure 4A). Changes in peak systolic and end diastolic renal blood velocity mirrored that of mean renal blood velocity, with peak systolic and end diastolic renal blood velocity decreasing during the cold pressor test (*P* < 0.01) and the magnitude of these decreases did not differ between pre- and post-exercise (*P* ≥ 0.10). RVR increased with face cooling (*P* < 0.01) and the extent of this increase did not differ between pre- and post-exercise (*P* = 0.12, Figure 4B).

## Discussion

Contrary to our hypotheses, our data demonstrate that prior aerobic or anaerobic exercise does not influence the extent of increases in RVR during sympathoexcitatory stimuli. Specifically, we have identified that 30 min of moderate intensity aerobic exercise that invoked ∼70% of age-predicted maximum heart rate did not affect the post-exercise renal vascular response to face cooling, a stimulus known to elicit sympathetically mediated increases in vascular resistance (Fisher et al., 2015; Schlader et al., 2016a, 2018; Johnson et al., 2018), including in the renal vasculature (Patel et al., 2013) (Figure 2). In a second study we found that the completion of a 30 s Wingate Anaerobic Test also did not affect the post-exercise renal vascular response to the cold pressor test, a stimulus known to increase vascular resistance secondary to sympathetic activation (Victor et al., 1987; Cui et al., 2010), including the renal vascular bed (Patel et al., 2013) (Figure 4). Collectively, these data indicate that prior moderate intensity aerobic exercise or brief maximal effort anaerobic exercise, to the extent studied in the present investigations, does not affect sympathetically mediated renal vasomotor responsiveness.

### Prior Aerobic Exercise Does Not Affect the Renal Vascular Response to Face Cooling (Study 1)

Orthostatic tolerance is often impaired following moderate intensity aerobic exercise (Halliwill et al., 2014). These observations are likely explained by a relative inability to increase vascular resistance during orthostasis (Lacewell et al., 2014). This reduces venous return, thereby compromising stroke volume and the ability to adequately maintain cardiac output (Krediet et al., 2004). The attenuated increase in vascular resistance is likely due to changes in the muscle vasculature, such that for a given sympathetic stimulus the increase in muscle vascular resistance is blunted (Halliwill et al., 1996a). However, to the best of our knowledge, a role for alterations in renal vascular control has not been discounted, despite that this vasculature contributes to blood pressure regulation during orthostasis (Bakris et al., 1986; Hirsch et al., 1989; Minson et al., 1999). Data in rats indicate that the relationship between blood pressure and renal sympathetic nerve activity is shifted downward following aerobic exercise (Miki et al., 2003). In humans, aerobic exercise induced elevations in RVR return to pre-exercise levels shortly after exercise (Pricher et al., 2004). This is supported by our data such that renal blood velocity and our estimate of RVR did not differ at baseline pre- versus post- aerobic exercise (Table 1). Based on the data presented herein (Table 1) and previous findings in humans (Pricher et al., 2004), together with the aforementioned study in rats (Miki et al., 2003), we speculate that a given level of renal sympathetic activity likely results in a higher RVR following aerobic exercise compared to before exercise. The reason for this is unknown, but may be related to the balance of circulating vasoactive factors, which likely more readily favor vasoconstriction in the renal vasculature. For instance, vasopressin and aldosterone both increase with moderate intensity aerobic exercise (Convertino et al., 1981; Freund et al., 1991). These vasoactive hormones could raise vascular resistance independent of sympathetic activation, particularly in the renal vasculature (Schmid et al., 1974, Schmidt et al., 2006). Notably, this contention is speculative. Therefore, more research is required to understand how prior exercise may affect the neuro-hormonal balance underlying the control of renal blood flow.

Our data also demonstrate that the magnitude of increases in RVR during face cooling are not affected by prior aerobic exercise (Figure 2). To the best of our knowledge, these findings are novel. Our data can likely be explained by the findings that the gain of the relation between renal sympathetic nerve activity and blood pressure are not affected by prior aerobic exercise (Miki et al., 2003). This suggests that the shift in the relationship between blood pressure and renal sympathetic nerve activity observed in rats (Miki et al., 2003), does not affect the capacity of the renal vasculature to respond to a sympathoexcitatory stimulus. Notably, the maintenance of blood pressure during orthostasis is dependent upon increasing sympathetic nerve activity (Cooke et al., 2009). Therefore, our data might suggest that an inability to vasoconstrict the renal vasculature following aerobic exercise is unlikely to contribute to post-exercise orthostatic intolerance.

It is important to note that our aerobic exercise paradigm did not elicit the expected post-exercise hypotension (Table 1). Thus, in the present study the expected reductions in TPR were offset by increases in cardiac output, which was almost entirely due to elevations in heart rate (Table 1). The reason for this observation is likely due to the duration and intensity of the aerobic exercise. In the present study subjects exercised at ∼70% of age-predicted maximum heart rate for 30 min, which is estimated to elicit 50–60% maximal oxygen uptake (Lounana et al., 2007). This exercise intensity and duration is slightly lower and shorter than that commonly used to study post-exercise hypotension (e.g., Halliwill et al., 1996a,b; Pricher et al., 2004; Wilkins et al., 2004; McCord et al., 2006). Furthermore, the magnitude of hypotension following aerobic exercise is dependent on the total work completed, and is not necessarily dependent on the exercise intensity or duration *per se* (Jones et al., 2007). Thus, we speculate that if our aerobic exercise was higher in intensity or longer in duration, post-exercise hypotension would have been observed. Importantly, however, we do not believe the lack of post-exercise hypotension following aerobic exercise invalidates our finding that the increase in RVR during sympathetic activation was not affected by prior aerobic exercise. For instance, impairments in blood pressure regulation during orthostasis have been observed following high intensity exercise, despite that hypotension was not observed before orthostasis (Lacewell et al., 2014; Sieck et al., 2016). It is possible that this prior observation is a function of the exercise intensity (discussed below), but it may also indicate that a hemodynamic or sympathetic stimulus (e.g., orthostasis) is required before alterations in physiological function can be observed (Halliwill et al., 2014).

Although it was not a primary outcome of our study, the greater fall in heart rate with face cooling following aerobic exercise is interesting (Figure 1). Face cooling stimulates cold afferents downstream of the trigeminal nerve, which simultaneously stimulates both the sympathetic and parasympathetic nervous systems (Schlader et al., 2016a). As observed in the present study (Figure 1), these reductions in heart rate do not significantly compromise cardiac output (Schlader et al., 2016a). Thus, blood pressure rises secondary to sympathetically mediated increases in vascular resistance (i.e., TPR) (Fisher et al., 2015; Schlader et al., 2016a) (Figure 1). The increase in heart rate during exercise is mediated by both the withdrawal and activation cardiac parasympathetic and sympathetic activity, respectively (White and Raven, 2014). The opposite occurs following aerobic exercise, as heart rate is restored toward pre-exercise levels (White and Raven, 2014). The findings presented herein suggest that the sensitivity to a parasympathetic stimulus is enhanced following moderate intensity aerobic exercise. We speculate that this is due to the greater prevailing parasympathetic drive during recovery from aerobic exercise and/or a greater end organ (cardiac) responsiveness, the latter of which could be related to the higher heart rates following aerobic exercise. Importantly, our findings are suggestive of the potential utility of face cooling as a tool to probe parasympathetic nervous system function (or dysfunction) (Johnson et al., 2017, 2018).

### Prior Anaerobic Exercise Does Not Affect the Renal Vascular Response to the Cold Pressor Test (Study 2)

The extent of post-exercise orthostatic intolerance is influenced by the intensity of the exercise (Mündel et al., 2015). To our knowledge, however, a study comparing prior aerobic and anaerobic exercise on post-exercise orthostatic tolerance has not been reported. That said, it is often speculated that high intensity (anaerobic) exercise elicits a greater incidence of pre-syncope (∼73% of the observations) during orthostasis compared to moderate intensity (aerobic) exercise (∼42% of the observations) (Halliwill et al., 2014). The mechanisms underlying such potential differences in orthostatic tolerance are likely the same between aerobic and anaerobic exercise, but they probably differ in magnitude (Halliwill et al., 2014). For instance, the magnitude of decreases in muscle vascular resistance post-exercise are likely greater and the extent by which muscle vascular resistance is increased during orthostasis is likely attenuated following anaerobic exercise compared to following aerobic exercise. That said, a potential contribution for alterations in cerebral blood flow regulation, occurring subsequent to hypocapnia (Lacewell et al., 2014; Sieck et al., 2016) and/or changes in autoregulation (Ogoh et al., 2007), is also possible. Nevertheless, a potential contribution for the renal vasculature in post-anaerobic exercise orthostatic intolerance has not been formally considered. As described above, a sympathetically mediated increase in RVR is an important contributor to blood pressure regulation during orthostasis (Bakris et al., 1986; Hirsch et al., 1989; Minson et al., 1999). Increases in RVR during exercise are intensity dependent (Grimby, 1965; Castenfors, 1977), and the resulting reductions in renal blood flow can be maintained for up to 60 min following high intensity exercise (Suzuki et al., 1996). This is supported by our data such that renal blood velocity was lower and estimated RVR was higher (*P* = 0.08) following anaerobic exercise (Table 2). These sustained reductions in renal perfusion observed following anaerobic exercise differ from what happens following moderate intensity exercise (Table 1) (Pricher et al., 2004). The reasons for these differences can likely be explained by the heightened sympathetic activation (Kenney and Zappe, 1994; Suzuki et al., 1996) and/or greater concentrations of circulating vasoactive hormones (e.g., vasopressin, aldosterone, etc.) (Convertino et al., 1981; Freund et al., 1991) following exercise, all of which are known to increase with increased exercise intensity. The renal circulation is also sensitive to changes in arterial carbon dioxide, such that hypocapnia reduces renal sympathetic nerve activity (Shirahata et al., 1985) and decreases RVR (Norman et al., 1970; Sharkey et al., 1998). Thus, we speculate that the moderate hypocapnia observed following anaerobic exercise in our study (Table 2) likely helped to maintain renal perfusion, such that if hypocapnia were not present the reductions in renal blood velocity (and increases in RVR) would have been greater. However, a role for changes in arterial carbon dioxide on renal vascular control following exercise remains to be fully elucidated.

Our data also demonstrate that the capacity to increase RVR during the cold pressor test is not affected by prior anaerobic exercise (Figure 4). As described above, it is likely that these findings can be explained by data demonstrating that the gain of the relation between renal sympathetic nerve activity and blood pressure are not affected by prior aerobic exercise (Miki et al., 2003). Thus, the data presented herein further this concept to anaerobic exercise such that, despite the relative vasoconstricted state, the capacity to increase vascular resistance in the renal vasculature during sympathetic stimulation was unaffected by prior anaerobic exercise.

### Methodological Considerations

There are a few methodological considerations that warrant discussion. First, we did not directly measure renal blood flow. Rather, we measured renal blood velocity using Doppler ultrasound. This enabled quantification of dynamic changes in an index of renal blood flow. This was deemed ideal to discern the effect of prior exercise on the renal vascular response to acute sympathetic stimulation, which would have been virtually impossible due to the relatively short data collection periods if we had used para-aminohippuric acid clearance, a traditional method for estimating renal blood flow in humans (Beierwaltes et al., 2013). That said, there are limitations associated with using Doppler ultrasound. For instance, renal blood flow is a function of artery diameter and blood velocity. Given the depth of the artery, it is not possible to accurately measure renal artery diameter using ultrasound. The diameter of the renal artery does not change during pharmacologically induced renal vasoconstriction (Marraccini et al., 1996). Thus, in the present study changes in renal blood velocity were interpreted to reflect changes in renal blood flow, as has been done previously (Wilson et al., 2007; Patel et al., 2013; Drew et al., 2017). However, it is acknowledged that we did not measure volumetric renal blood flow. Moreover, due to potential differences in the insonation location and/or angle, it is possible that the test–retest reliability of the Doppler ultrasound measurement of renal blood velocity is poor. To overcome this limitation, controls were put in place to ensure our insonation location and angle were the same pre- and post- exercise. Furthermore, our data during face cooling and the cold pressor test were primarily analyzed as the absolute change from baseline. Nevertheless, conclusions associated with baseline measurements (e.g., Tables 1,2) should be made with caution. However, it is notable that the 6% reduction in renal blood velocity observed following anaerobic exercise (Table 2) is outside of the variation in the measurement of our sonographer (∼4%). Second, the data presented in Studies 1 and 2 were obtained from two independent studies that used different sympathoexcitatory stimuli and a different cohort of subjects. Given these differences in study design, direct comparisons between the two Studies were not made. Therefore, the direct comparative effects of aerobic versus anaerobic exercise on the renal vascular response to sympathetic stimulation remains unknown. Third, we did not directly measure any indices of sympathetic activation (e.g., muscle sympathetic nerve activity) and it is not possible to measure renal sympathetic nerve activity in humans. Therefore, it is unknown if the magnitude of the whole-body and/or renal sympathetic response invoked by face cooling (Study 1) and the cold pressor test (Study 2) was the same pre- versus post-exercise. Fourth, exercise induced body fluid losses and/or elevations in body temperature may play a role in post-exercise hypotension and orthostatic intolerance (Halliwill et al., 2013, 2014; Meade et al., 2018). However, we did not measure any aspects of body fluid status (e.g., changes in body weight or plasma volume) or body temperature (e.g., core temperature). Thus, a potential contribution of these factors to the renal vascular response following exercise remains unknown. Fifth, we tested both males and females and we did not control for menstrual cycle phase in our female subjects. Notably, we are underpowered to conduct a formal analysis between males and females. There is some evidence that the incidence of post-exercise orthostatic intolerance is lower in females (Halliwill et al., 2014). Thus, it is possible that the renal vascular response to sympathetic stimulation following aerobic or anaerobic exercise may differ between males and females, and across the menstrual cycle. Finally, we tested the renal vascular response to hypertension-invoking sympathetic stimuli in the supine position. This enabled the measurement of dynamic changes in RVR using Doppler ultrasound. However, whether our findings would differ if they were obtained during orthostasis or with unloading of the baroreceptors, as occurs with lower body negative pressure, is unknown.

### Perspectives

Our studies provide unique insights into the recovery of the cardiovascular and renal systems after aerobic or anaerobic exercise. This is important because the post-exercise recovery period presents a key window of opportunity that may be used to promote training adaptations (Luttrell and Halliwill, 2015). This may be particularly important as it relates to plasma volume, whereby post-exercise hypotension appears to be a key determinant of training invoked plasma volume expansion (Hayes et al., 2000). Moreover, our studies also have the potential to inform the development of countermeasures to protect against syncope following exercise. Collectively, our data indicate that an inability to increase resistance in the renal vasculature following aerobic or anaerobic exercise is unlikely to contribute to post-exercise orthostatic intolerance. Thus, interventions aimed toward augmenting RVR post-exercise are unlikely to be affective at alleviating the incidence of orthostatic intolerance following aerobic or anaerobic exercise. Rather, such interventions should selectively target the muscle vasculature (McCord et al., 2008) or venous return more generally, as can be augmented by respiratory impedance (Lacewell et al., 2014).

## Conclusion

The present study demonstrates that 30 min of moderate intensity aerobic exercise or 30 s of maximal effort anaerobic exercise does not affect the capacity to increase RVR during sympathetic stimulation following exercise.

## Author Contributions

ZS, CC, NV, and BJ conceptualized the studies. CC, JB, EG, NV, and PL collected the data. ZS, CC, JB, EG, and PL analyzed the data. ZS, CC, NV, and BJ contributed to data interpretation. ZS drafted the manuscript. All authors approved the finalized manuscript.

## Conflict of Interest Statement

PL is a consultant for Mindray North American Ultrasound. The remaining authors declare that the research was conducted in the absence of any commercial or financial relationships that could be construed as a potential conflict of interest.
